# Autotaxin Induces S1P/S1PR1 Signaling to Affect Th17/Treg Cell Balance and Exacerbate Intestinal Inflammation in Colitis

**DOI:** 10.3390/ijms27062861

**Published:** 2026-03-21

**Authors:** Siqi Xiao, Kaixin Peng, Congxin Li, Yuanyuan Long, Hongbing Yu, Suhong Xia, Qinghai Tan, Qin Yu

**Affiliations:** 1 Department of Gastroenterology, Tongji Hospital of Tongji Medical College, Huazhong University of Science and Technology, Wuhan 430030, China; xsq_17@163.com (S.X.);; 2 Institute of Liver and Gastrointestinal Diseases, Tongji Hospital of Tongji Medical College, Huazhong University of Science and Technology, Wuhan 430030, China; 3 Department of Microbiology, Molecular Genetics and Immunology, University of Kansas Medical Center, Kansas City, KS 66160, USA; hyu3@kumc.edu

**Keywords:** autotaxin, sphingosine-1-phosphate, Th17/Treg cell balance, intestinal immunity, colitis

## Abstract

Abnormal intestinal mucosal immunity plays a crucial role in ulcerative colitis (UC). Autotaxin (ATX) can promote T cell migration and was reported to have a regulatory effect on Th17 cells, while sphingosine-1-phosphate (S1P) and its receptors (S1PRs) modulate Th17/Treg balance and inflammation, with S1PR modulators approved for UC. ATX can catalyze sphingosylphosphorylcholine (SPC) to produce S1P; however, the relationship between ATX and S1P/S1PRs in UC is unclear. Understanding the role of ATX-S1P/S1PRs in intestinal immunity can provide new treatment strategies for intestinal inflammatory diseases. Both UC patients and DSS-induced colitic mice showed significantly increased levels of ATX and S1P compared with healthy controls. ATX inhibitor PF8380 treatment led to reduced levels of S1P/S1PRs in colitic mice. Consistent with this, the S1PR antagonist etrasimod was able to alleviate ATX-induced intestinal inflammation, as well as partially restore ATX-induced Th17/Treg imbalance in MLNs and the spleen. In HT-29 and Raw246.7 cells, ATX treatment led to enhanced expression of S1P/S1PRs, with S1PR1 being the most significant. Furthermore, S1PR1 mediates the effect of ATX on Th17/Treg cell differentiation and function in vivo. Therefore, ATX affects the differentiation and function of Th17/Treg cells through S1P/S1PR1 signaling, increased ATX expression leading to Th17/Treg cell imbalance, intestinal mucosal immune dysfunction, and exacerbating intestinal inflammation.

## 1. Introduction

Ulcerative colitis (UC) is a complex and chronic non-specific inflammatory disease of the intestine with unknown etiology. UC patients often have recurrent and persistent inflammatory conditions in their intestines that require lifelong treatment. As the disease progresses, it can lead to intestinal disabling changes, including intestinal perforation, obstruction, and colon cancer, which seriously affect the quality of life of patients and bring economic burden [1]. Recently, the global incidence rate of UC has been on the rise. In East Asia, including China, due to population growth and aging, the burden of UC will continue to rise in the next 25 years [2]. At present, the therapeutic drugs for UC mainly include aminosalicylic acid, glucocorticoids, immunosuppressants, and biologics. Notably, biologics represented by TNF-α monoclonal antibodies have achieved good clinical efficacy. However, TNF-α monoclonal antibody treatment did not reduce the hospitalization rate and intestinal resection rate of UC [3], and often faced the problem of immune failure in some patients [4,5]. Therefore, an in-depth exploration of the pathogenesis of UC is needed, potentially leading to the identification and development of novel therapies for UC.

The pathogenesis of UC is not yet clear, but it is widely believed that the interaction of environmental, genetic, and gut microbiota factors can cause immune dysfunction of the intestinal mucosa and disrupt the intestinal epithelial barrier, ultimately leading to the occurrence and development of intestinal inflammation [6]. In recent years, people have gradually paid attention to the important role of intestinal mucosal immune dysfunction in intestinal inflammation, and the impact of Th17/Treg cell imbalance has been confirmed by previous studies [7,8,9]. Th17 and Treg cells exhibit completely opposite regulation in differentiation and function. The differentiation direction of immature CD4^+^T cells varies in the presence of different cytokines. IL-6 and TGF-β are considered essential cytokines for Th17 differentiation, and when IL-6 is lacking, T cells differentiate towards Treg [10]. The balance of Th17/Treg cells is a key factor in ensuring the immune homeostasis of the body. Once the balance is disrupted, Th17/Treg cells participate in the progression of various pathological states through the secretion of cytokines. Studies have reported that the imbalance of Th17/Treg cells exacerbated the progression of inflammation in UC [11,12]. Therefore, methods aimed at maintaining intestinal Th17/Treg cell balance are potential therapeutic strategies for UC. In order to develop this therapy, a deeper understanding of the regulatory mechanisms of intestinal Th17/Treg cell balance is necessary.

Autotaxin (ATX) is a member of the extracellular nucleotide pyrophosphate/phosphodiesterase (ENPPs) family. Two recent studies have confirmed that ATX expression is increased in acute and chronic colitis, and inhibiting ATX activity can improve intestinal inflammation [13,14]. Our previous research has confirmed that both dextran sulfate sodium salt (DSS)-induced colitic mice and UC patients exhibited increased infiltration of inflammatory cells, including macrophages, in the colon mucosa, and upregulation of ATX expression in the colon mucosa tissue and serum. In the DSS-induced colitis model, mice treated with an ATX inhibitor showed decreased infiltration of inflammatory cells and production of inflammatory factors [15]. Intriguingly, we found ATX itself can induce colonic inflammation in mice, resulting in colon shortening, an impaired epithelial barrier and increased inflammatory cell infiltration [16]. These findings suggest that ATX may be an important factor in the pathogenesis of UC [15]. ATX has been shown to participate in various immune regulations by catalyzing the production of extracellular lysophosphatidic acid (LPA) [17,18]. Studies have shown that blocking the ATX-LPA axis can alleviate DSS-induced chronic colitis in mice by inhibiting Th17 cell differentiation [14]. Other studies have shown that ATX can promote T cell migration, thereby recruiting T cells to the lesion site to induce inflammation [19,20]. However, how ATX regulates the balance of intestinal Th17/Treg cells needs further investigation.

ATX can degrade sphingosylphosphocholine (SPC) to Sphingosyl-1-phosphate (S1P) [21]. S1P is the sphingolipid ligand of G protein-coupled receptors (S1PRs1-5), which control the migration of lymphocytes from lymphoid organs. The interaction of S1P/S1PRs can also lead to the internalization of S1PRs, thereby reducing the release of lymphocytes from lymphoid tissue to circulation [22,23]. Research has shown that S1P promotes the differentiation of CD4^+^ T cells into Th1/Th17 cells [24], but inhibits the generation of extrathymic cells and natural Treg cells [25]. And Th17 cells are excreted from the intestine in an S1PR1-dependent manner [26]. Therefore, we speculate that S1P/S1PRs are involved in regulating the balance of Th17/Treg cells and affect the development of diseases. The intestinal tissue biopsy of IBD patients contains high levels of pro-inflammatory sphingolipids as well as S1PR1, S1PR2, and S1PR4. Inhibiting the binding of S1P and its ligands can effectively reduce the migration of lymphocytes to the site of intestinal inflammation [27]. At present, many S1P ligand antagonists have completed the clinical trial stage of IBD treatment and have shown good efficacy in UC patients. The selective S1PR1, S1PR4, and S1PR5 antagonist etrasimod (APD334) is a novel oral small molecule S1P receptor modulator, which was launched in the United States in October 2023 and has shown good therapeutic effects mainly for patients with moderate to severe active UC [28]. The new generation of oral small molecule S1P modulators are expected to become another milestone drug after monoclonal antibodies, such as Infliximab, Adalimumab and Vedolizumab, and have the characteristics of convenient oral administration, short half-life, and no immunogenicity. These S1P modulators have great therapeutic potential for UC, and may alleviate the problem of the poor efficacy of biological agents. However, whether ATX affects Th17/Treg cell balance through S1P/ S1PR is currently unclear.

In this study, we observed a potential correlation between ATX and S1P/S1PRs in UC patients and mice. Further research revealed that etrasimod could alleviate ATX-induced colitis in mice and inhibit the regulatory effect of ATX on the balance between Th17 and Treg cells. In vitro studies using HT-29 cells and Raw264.7 cells confirmed the regulatory effect of ATX on S1P/S1PR1. Moreover, ATX could enhance the secretion of pro-inflammatory cytokines, inhibit the secretion of anti-inflammatory cytokines, and regulate the expression of transcription factors related to Th17/Treg cells. Notably, silencing S1PR1 in cells could suppress the effects of ATX. In vivo experiments with selective S1PR1 antagonists and agonists demonstrated that S1PR1 mediated the regulatory effect of ATX on the differentiation and function of Th17/Treg cells, as well as the pro-colitis effect of ATX.

## 2. Results

### 2.1. The Levels of ATX and S1P/S1PRs Are Increased During Colitis

**Figure 1:**
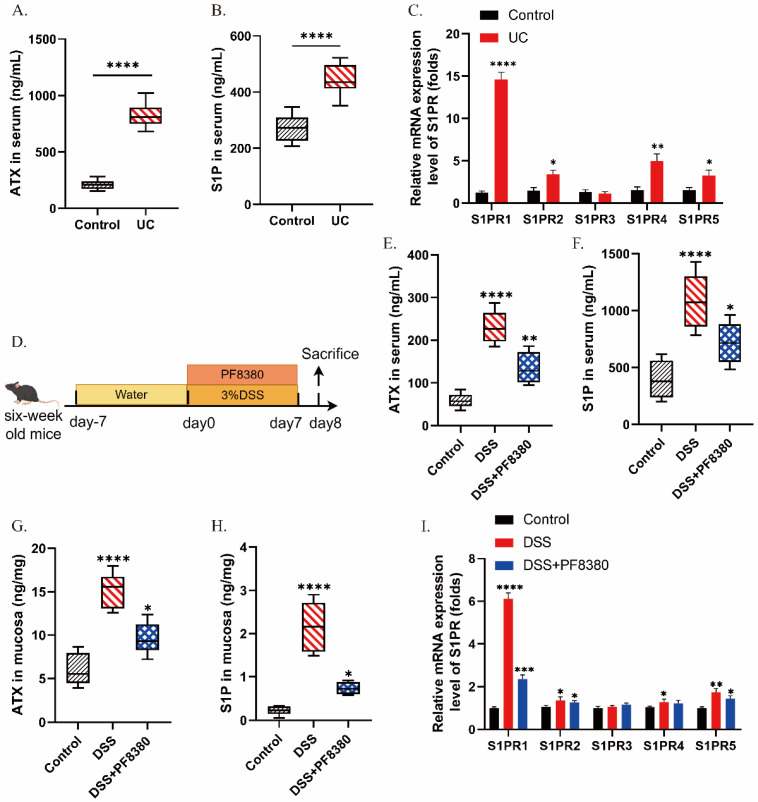
The levels of S1P, ATX, and colonic S1PRs in UC patients and DSS colitis mice. (**A**,**B**) The secretion level of ATX and S1P in the serum of UC patients and healthy volunteers was detected by ELISA (*n* = 10). (**C**) The relative mRNA expression of S1PRs in the colonic tissues of UC patients and healthy volunteers (*n* = 6). (**D**) Mice freely drank 3% DSS daily to establish an acute colitis model. The DSS + PF8380 group was simultaneously given PF8380 by gavage for 7 days (*n* = 6). (**E**,**F**) The secretion levels of ATX and S1P in the serum of mice in each group were detected by ELISA. (**G**,**H**) The secretion levels of ATX and S1P in the colonic tissues of mice in each group were detected by ELISA. (**I**) The relative mRNA expression of S1PRs in the colonic tissues of mice in each group. The data are presented as mean ± standard error. Compared with the control group, * *p* < 0.05, ** *p* < 0.01, *** *p* < 0.001, **** *p* < 0.0001.

To explore whether there is a correlation between ATX and S1P/S1PRs during colitis and to lay the foundation for subsequent research on their regulatory mechanisms, we conducted relevant experiments. In the human study, we recruited 10 UC patients and 10 healthy volunteers, and collected their serum samples to measure ATX and S1P levels. Meanwhile, colon tissue samples were collected from these participants to detect the expression levels of S1PR1, 2, 4, and 5. The results showed that serum ATX and S1P levels in UC patients were significantly higher than those in healthy controls (Figure 1A,B). Consistent with the elevation of its converting enzyme ATX, serum SPC levels were also elevated in UC patients compared to healthy volunteers (Supplementary Figure S1A), suggesting a systemic activation of sphingolipid metabolism during UC inflammation. In addition, the levels of S1PR1, 2, 4, and 5 in the colon tissue of UC patients were elevated compared to healthy controls, with the most significant increase observed in S1PR1 (Figure 1C). Subsequently, to further verify the correlation between ATX and S1P/S1PRs in colitis, we established a DSS-induced colitis model in mice. Specifically, mice were allowed to freely drink a 3% DSS solution for 7 days to induce colitis (Figure 1D). After the induction period, serum and colon tissue samples were collected from the mice. The experimental results revealed that compared to the control group, the content of ATX and S1P in the serum and colon tissue of the DSS-induced colitis mice group was increased. Additionally, the mRNA expression levels of S1PR1, 2, 4, and 5 were upregulated, with the most significant increase in S1PR1 (Figure 1E–I). These findings suggest a strong correlation between ATX content and the secretion of S1P as well as the expression of S1PRs during colitis.

### 2.2. S1PR Modulator Etrasimod Alleviates ATX-Induced Colonic Inflammation in Mice

**Figure 2:**
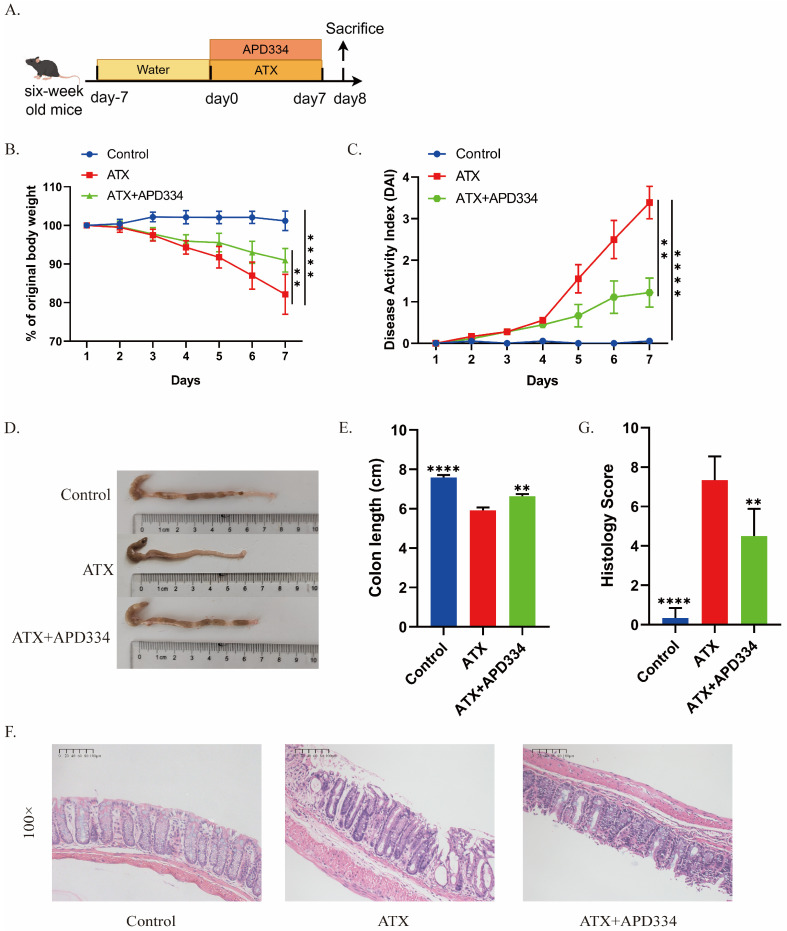
Etrasimod alleviates ATX-induced colonic inflammation. (**A**) Mice were given ATX by gavage daily to establish an acute colitis model. The ATX + APD334 group was simultaneously given APD334 by gavage for 7 days. (**B**) The changes in body weight of the mice. (**C**) The Disease Activity Index (DAI) score. (**D**,**E**) The colonic lengths of the mice in each group. (**F**) Hematoxylin and eosin (HE) staining of the distal colon of the mice in each group. (**G**) The histopathological scores of the distal colon tissues of the mice in each group. The data are presented as mean ± standard error (n = 6). Compared with the ATX group, ** *p* < 0.01, **** *p* < 0.0001.

Etrasimod (APD334) is an oral small molecule selective S1P receptor modulator (S1PR1, 4, 5 antagonist) that has shown good therapeutic effects on patients with moderate to severe active UC in clinical practice. It has been approved for clinical use by the FDA [28]. In previous studies, we demonstrated that ATX treatment induced colonic inflammation in mice [16]. To further verify the relationship between ATX and S1P/S1PRs, etrasimod was administered orally to ATX-treated mice daily. As shown in Figure 2A, C57BL/6 mice were divided into three groups: control group, ATX group, and ATX + APD334 group. We monitored the clinical symptoms of these mice, including body weight loss, colon length and their Disease Activity Index (DAI). Compared with the control group, ATX-treated mice showed significant body weight loss, increased DAI scores, and shortened colon length (Figure 2B–E). Histopathological examination showed that after ATX treatment, the distal colon exhibited significant infiltration of inflammatory cells, loss of goblet cells, and crypt damage (Figure 2F,G). Notably, etrasimod treatment led to a reduction in weight loss, a decrease in the DAI scores, an increase in colonic length, and a decrease in the pathological score in ATX-treated mice (Figure 2B–G). The results suggest that the pro-inflammatory effect of ATX on colonic inflammation may be mediated by S1P receptors.

### 2.3. The S1P Receptor Modulator Etrasimod Regulates Th17/Treg Cell Balance in the Spleen and Mesenteric Lymph Nodes of ATX-Treated Mice

**Figure 3:**
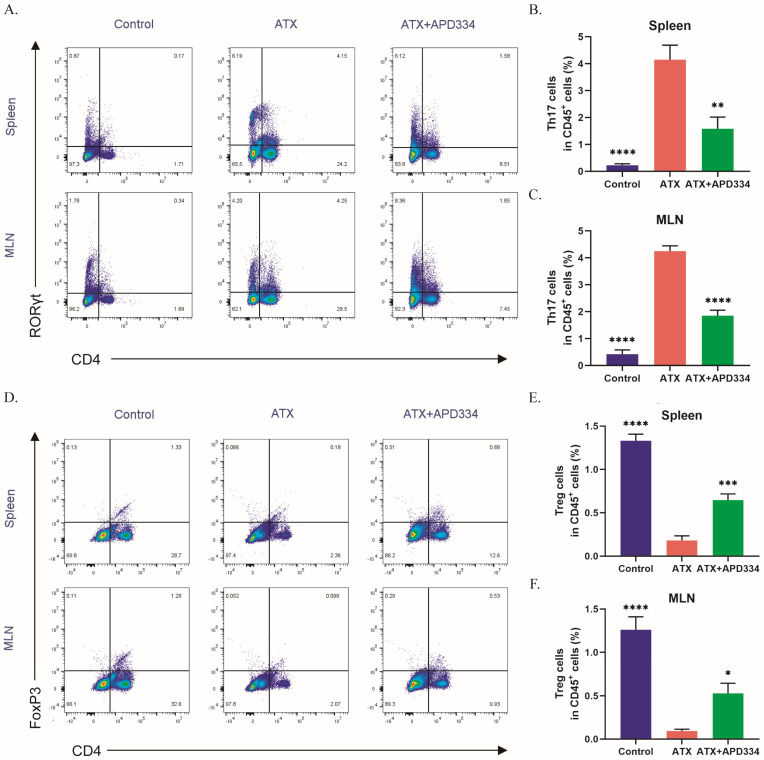
Etrasimod regulates the balance of Th17/Treg cells in the spleen and mesenteric lymph nodes of ATX colitis mice. (**A**) Flow cytometry was used to determine the percentage of CD4^+^RORγt^+^ (Th17) cells in the spleen and mesenteric lymph nodes. (**B**,**C**) Statistical analysis of the ratio of Th17 cells among CD45^+^ cells in the spleen and mesenteric lymph nodes. (**D**) Flow cytometry was used to determine the percentage of CD4^+^Foxp3^+^ (Treg) cells in the spleen and mesenteric lymph nodes. (**E**,**F**) Statistical analysis of the ratio of Treg cells among CD45^+^ cells in the spleen and mesenteric lymph nodes. The data are presented as mean ± standard error (n = 6). Compared with the ATX group, * *p* < 0.05, ** *p* < 0.01, *** *p* < 0.001, **** *p* < 0.0001.

S1P/S1PRs have been shown to participate in the regulation of Th17/Treg cell balance [29]. And there have been studies reporting the regulatory effect of ATX on Th17 cells [14]. Since ATX can degrade SPC to S1P, we speculated that ATX could regulate Th17/Treg cell balance through S1P/S1PRs, thereby exerting pro-inflammatory effects. We used flow cytometry to quantify the percentages of Th17 and Treg cells in the spleen and mesenteric lymph nodes of the aforementioned mice groups to explore the effect of etrasimod on Th17/Treg cell balance. Figure 3A–C show the percentage of CD4^+^RORγt^+^ (Th17) cells and Figure 3D–F show the percentage of CD4^+^Foxp3^+^ (Treg) cells in CD45^+^ cells in the mouse spleen and mesenteric lymph nodes. Compared with the control group, the ATX-treated group showed an increase in Th17 cell level and a decrease in Treg cell level in the spleen and mesenteric lymph nodes, while etrasimod treatment was able to reverse the effect of ATX on Th17 and Treg cell levels (Figure 3A–F). These data demonstrate that the S1P receptor modulator can partially reverse the regulatory effects of ATX on Th17/Treg cell balance.

### 2.4. S1PR1 Induced by ATX Mediates the Effect of ATX in Colitis Mice

**Figure 4:**
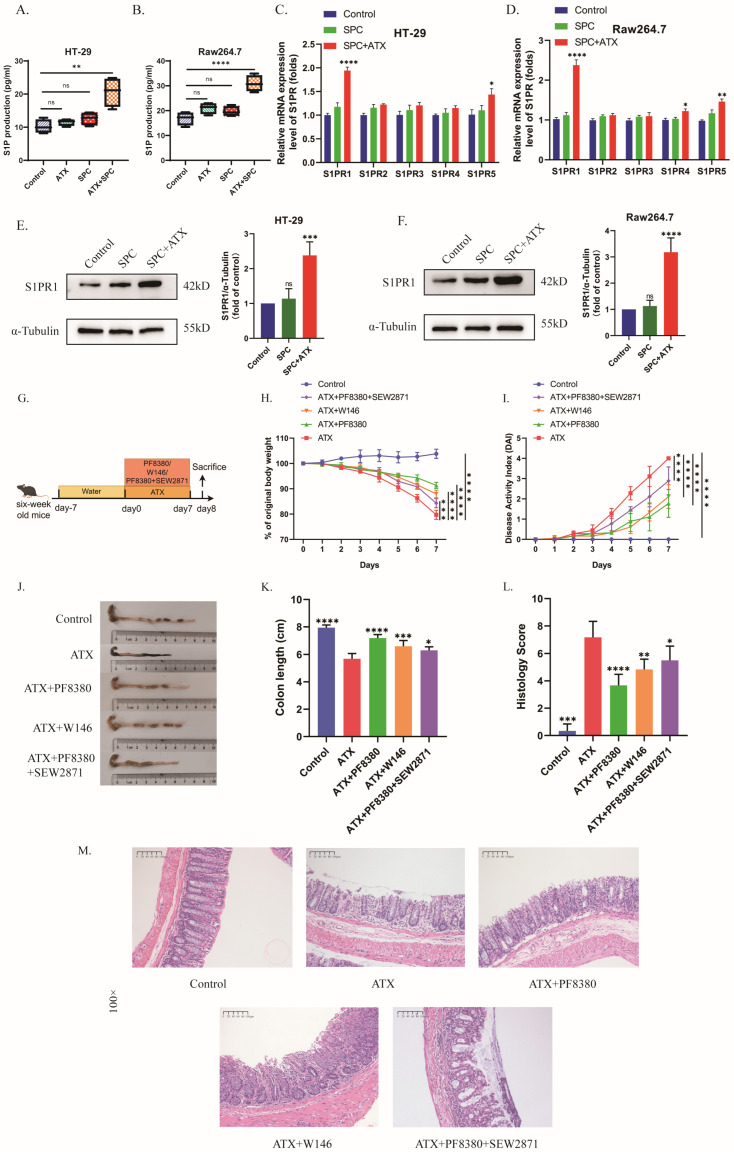
ATX promotes the secretion of S1P and the expression of S1PRs in cells, and S1PR1 mediates the effect of ATX in colitis mice. (**A**,**B**) The secretion levels of S1P in the supernatants of HT-29 cells and Raw264.7 cells were detected by ELISA. (**C**,**D**) The mRNA levels of S1PRs in HT-29 cells and Raw264.7 cells were detected by qRT-PCR. (**E**,**F**) The protein levels of S1PR1 in HT-29 cells and Raw264.7 cells were detected by Western blot. The data are presented as mean ± standard error. Compared with the control group, * *p* < 0.05, ** *p* < 0.01, *** *p* < 0.001, **** *p* < 0.0001. (**G**) Experimental design. (**H**) The changes in body weight of the mice. (**I**) The Disease Activity Index (DAI) score. (**J**,**K**) The colonic lengths of the mice in each group. (**L**) The histopathological scores of the distal colon tissues of the mice in each group. (**M**) Hematoxylin and eosin (HE) staining of the distal colon of the mice in each group. The data are presented as mean ± standard error (n = 6). Compared with the ATX group, * *p* < 0.05, ** *p* < 0.01, *** *p* < 0.001, **** *p* < 0.0001.

The results in Figure 1 demonstrated the correlation between ATX and S1P, as well as S1PRs (especially S1PR1). We further validated that at the cellular level. Selecting HT-29 (human colon cancer cells) and Raw264.7 (mouse macrophage-like cells) as the research subjects, three different treatments were performed on the cells: no special treatment (control group); SPC treatment only (SPC group); and SPC and ATX treatment simultaneously (SPC + ATX group). The results showed that simultaneous treatment with SPC and ATX significantly promoted the secretion of S1P in cells, while SPC alone had little effect on S1P secretion (Figure 4A,B). The qRT-PCR results showed that the promotion of S1PR1 expression by ATX was significant in both HT-29 and Raw264.7 cells (Figure 4C,D). The Western blot results further confirmed the promoting effect of ATX on S1PR1 expression (Figure 4E,F).

We further validated that S1PR1 mediated the pro-inflammatory effect of ATX in mice. The S1PR1-selective antagonist W146 and selective agonist SEW2871 were used to intervene in ATX-treated mice. C57BL/6 mice were divided into five groups (Figure 4G): control group, ATX group, ATX + PF8380 group, ATX + W146 group, and ATX + PF8380 + SEW2871 group. The results showed that W146 could alleviate the colonic inflammatory state of ATX-treated mice, and its effect was similar to that of the ATX inhibitor PF8380 (Figure 4H–M). In addition, the treatment of SEW2871 could partially reverse the alleviating effect of PF8380 on ATX-induced colitis (Figure 4H–M). These data indicate that S1PR1 could mediate the pro-inflammatory effect of ATX on mice.

**Figure 5:**
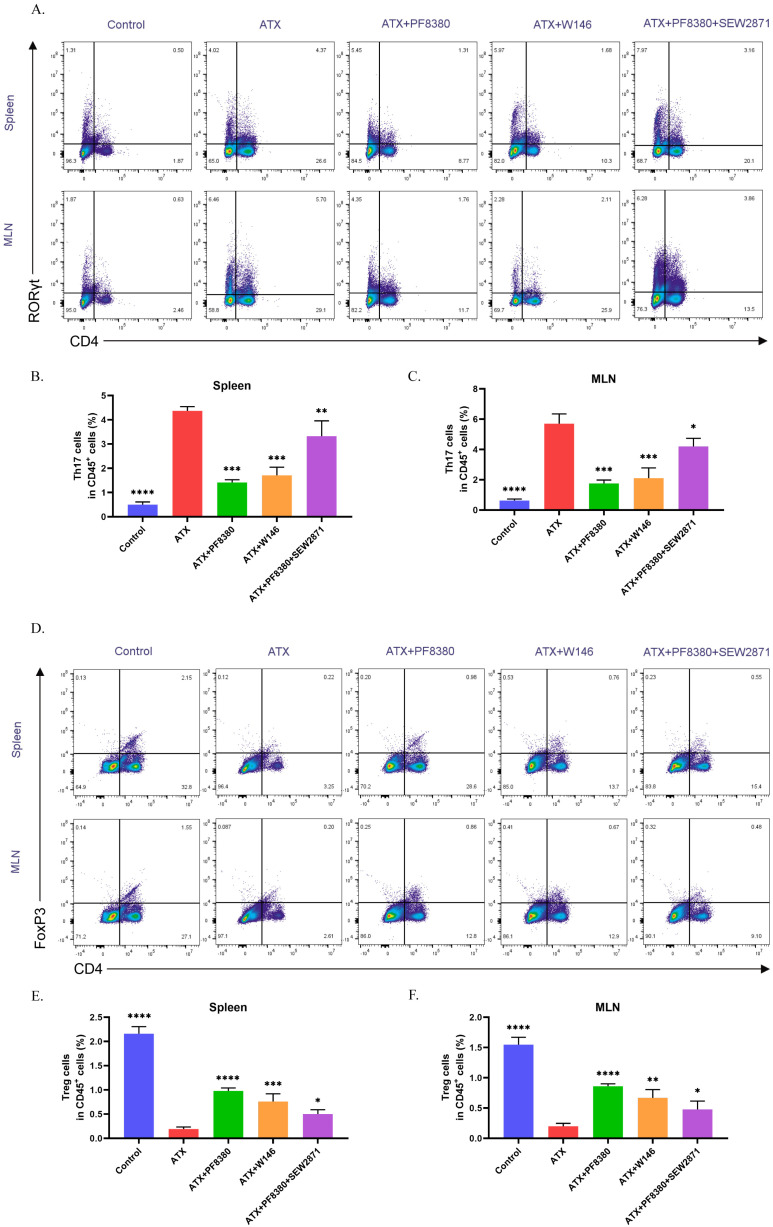
Selective antagonists and agonists of S1PR1 alter the ratio of Th17/Treg cells in ATX colitis mice. (**A**) Flow cytometry was used to determine the percentage of CD4^+^RORγt^+^ (Th17) cells in the spleen and mesenteric lymph nodes. (**B**,**C**) Statistical analysis of the ratio of Th17 cells among CD45^+^ cells in the spleen and mesenteric lymph nodes. (**D**) Flow cytometry was used to determine the percentage of CD4^+^Foxp3^+^ (Treg) cells in the spleen and mesenteric lymph nodes. (**E**,**F**) Statistical analysis of the ratio of Treg cells among CD45^+^ cells in the spleen and mesenteric lymph nodes. The data are presented as mean ± standard error (n = 6). Compared with the ATX group, * *p* < 0.05, ** *p* < 0.01, *** *p* < 0.001, **** *p* < 0.0001.

We used flow cytometry to detect the percentages of Th17 and Treg cells in the spleen and mesenteric lymph nodes of the five groups of mice mentioned above, and found that, compared with the control group, ATX treatment led to an increase in Th17 levels and a decrease in Treg levels in the spleen and mesenteric lymph nodes of mice, and the ATX inhibitor PF8380 was able to reverse this effect (Figure 5A–F). W146 exhibited a similar effect to PF8380, while SEW2871 could weaken the effect of PF8380. These data indicate that the effect of ATX on Th17/Treg cell balance in mice could be mediated by S1PR1.

### 2.5. S1PR1 Mediates the Effect of ATX on Th17/Treg Cell Differentiation and Function In Vivo

**Figure 6:**
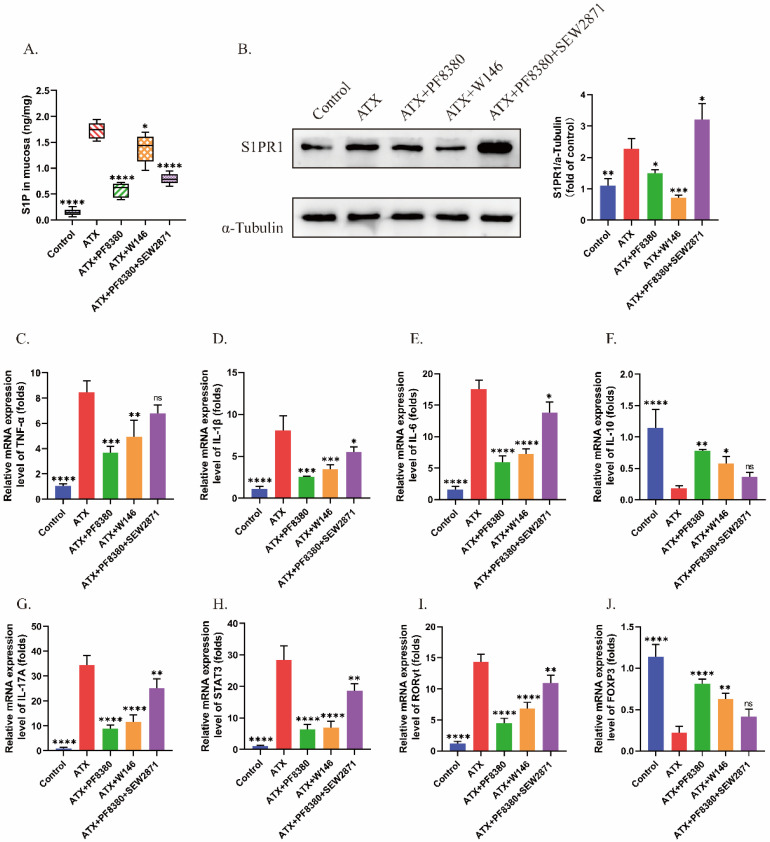
Selective antagonists and agonists of S1PR1 affect ATX’s regulation of cytokines in mouse colonic tissues. (**A**) The secretion level of S1P in the colonic tissues of mice in each group was detected by ELISA. (**B**) The protein level of S1PR1 in the colonic tissues was detected by Western blot. (**C**–**J**) The mRNA levels of TNF-α, IL-1β, IL-6, IL-10, IL-17A, STAT3, RORγt and FOXP3 in the colonic tissues of mice in each group were detected by qRT-PCR. The data are presented as mean ± standard error (n = 6). Compared with the ATX group, * *p* < 0.05, ** *p* < 0.01, *** *p* < 0.001, **** *p* < 0.0001.

Furthermore, we investigated the effects of ATX-S1P/S1PR1 on Th17/Treg cell differentiation and function in mice. ATX gavage increased the S1P content in mouse colon tissue, while the ATX inhibitor inhibited the S1P production induced by ATX (Figure 6A). Western blot results showed that both the ATX inhibitor and S1PR1 selective antagonist inhibited the expression of S1PR1 in the colon tissue of ATX-treated mice, while the S1PR1 selective agonist reversed the effect of the ATX inhibitor (Figure 6B).

The mRNA expression levels of cytokines in mouse colon tissue were detected by qRT-PCR. ATX exhibited a pro-secretion effect on pro-inflammatory factors and an inhibitory effect on anti-inflammatory factors (Figure 6C–G). In addition, ATX affected the expression of Th17/Treg cell transcription regulatory factors STAT3, RORγt, and FOXP3 (Figure 6H–J). The introduction of a S1PR1 selective antagonist resulted in a decrease in the expression levels of Th17 cell representative functional cytokine IL-17A, as well as the transcription regulatory factors STAT3 and RORγt in the colon tissue of ATX-treated mice, and an increase in the expression of Treg cell representative functional cytokine IL-10 and transcription regulatory factor FOXP3, similar to the effect of the ATX inhibitor (Figure 6C–J). The S1PR1 selective agonist weakened the effect of the ATX inhibitor on the expression of cytokine TNF-α, IL-1β, IL-6, IL-10, IL-17A, STAT3, RORγt and FOXP3 (Figure 6C–J). Altogether, S1PR1 could mediate the regulatory effect of ATX on Th17/Treg cell differentiation and function in mice.

### 2.6. S1PR1 Selective Antagonist Combined ATX Inhibitor Alleviates Colon Inflammation in DSS-Induced Colitis

**Figure 7:**
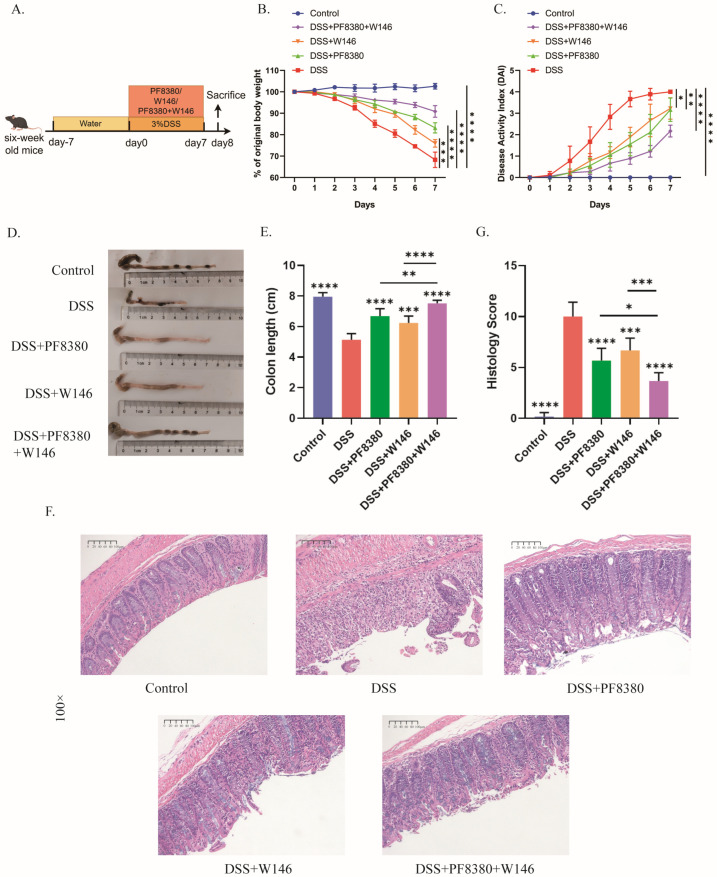
The combination of ATX inhibitor and selective S1PR1 antagonist significantly alleviates DSS-induced colonic inflammation in mice. (**A**) Experimental design. (**B**) Changes in the body weight of the mice. (**C**) Disease Activity Index (DAI) score. (**D**,**E**) The colonic lengths of the mice in each group. (**F**) Hematoxylin and eosin (HE) staining of the distal colon of the mice in each group. (**G**) Histopathological scores of the distal colon tissues of the mice in each group. The data are presented as mean ± standard error (n = 6). Compared with the DSS group, * *p* < 0.05, ** *p* < 0.01, *** *p* < 0.001, **** *p* < 0.0001.

Our previous studies have confirmed that the ATX inhibitor PF8380 can alleviate intestinal inflammation in DSS colitis mice [15]. At the end of this study, we evaluated the therapeutic effect of an S1PR1 selective antagonist in DSS colitis mice. We divided C57BL/6 mice into five groups (Figure 7A): control group, DSS group, DSS + PF8380 group, DSS + W146 group, and DSS + PF8380 + W146 group. According to observations, compared with the DSS group, the DSS + W146 group showed amelioration in weight loss, colon shortening, and high DAI scores, achieving similar results as the DSS + PF8380 group (Figure 7B–E). The histopathological analysis of the distal colon tissue also confirmed the anti-inflammatory effect of W146 on DSS-induced colitis (Figure 7F–G). In addition, we applied a combination of the ATX inhibitor and S1PR1 selective antagonist to DSS colitis mice and were surprised to find that the combination of the two drugs could further alleviate colitis in mice, achieving a better effect than the single use of the two drugs (Figure 7B–G).

## 3. Discussion

ATX is a secreted glycoprotein encoded by the ENPP2 gene, which is a key enzyme for extracellular LPA production and exists in various biological fluids and tissues [30]. It participates in various physiological processes, including reproduction, central nervous system development, immunity, inflammation, and lipid metabolism [31]. Meanwhile, ATX is also associated with various pathological conditions, especially inflammation and tumor-related diseases [32]. ATX maintains inflammation by inducing the production of pro-inflammatory cytokines. Multiple studies have reported an increase in ATX levels in DSS-induced colitis, and blocking ATX can alleviate colitis in mice through the inhibition of Th17 cell differentiation [13,15,33]. ATX is believed to primarily exert effects through LPA, thereby participating in the inflammation and immune regulation of IBD [34]. We have previously shown that UC plasma LPA is likewise elevated and reduced by ATX inhibition [15]; the present work therefore focuses on the less-explored ATX-S1P branch, while not excluding the ATX-LPA pathway. However, as a secretory enzyme with hemolytic phospholipase D activity, ATX has been reported to be able to degrade SPC and convert it into S1P. Although sphingosine kinases (SPHK1/2) can phosphorylate free sphingosine to generate S1P, Snider et al. have already documented up-regulated SPHK1 in DSS colitis [35]; repeating these assays would not alter the principal conclusion of the present work. Under our serum-free cell-culture conditions, ATX alone (without exogenous SPC) failed to raise S1P, implying that SPHK1-mediated conversion of endogenous sphingosine was minimal. Collectively, we propose that ATX-mediated cleavage of SPC constitutes a hitherto under-appreciated route that synergistically amplifies local S1P in inflamed mucosa. Research has confirmed the role of the ATX-S1P pathway in the development of tumors related to tuberous sclerosis [36]. However, there are currently few reports on the role of the ATX-S1P pathway in IBD. In our study, an analysis of serum levels of ATX and S1P revealed a concomitant increase in these molecules in the peripheral blood of UC patients compared to healthy volunteers. Subsequent investigation in a DSS-induced colitis mouse model demonstrated that this elevation is not exclusive to humans, as similar increases in ATX and S1P levels were observed in the mouse model. Further experimentation uncovered a corresponding upregulation of S1P receptors within the colonic tissue of both UC patients and affected mice. These findings collectively point to a potential correlation between ATX and S1P in the context of colitis, suggesting a mechanistic link that may be pivotal in the pathogenesis of intestinal inflammation. This association warrants further exploration to elucidate the precise nature of their interaction and its implications for therapeutic intervention in UC. Future kinetic modeling with stable-isotope-labeled SPC and sphingosine will be required to quantify the exact contribution of each biosynthetic route under steady-state and inflammatory conditions.

S1P is an effective signaling molecule involved in inflammation and proliferation, and many studies have emphasized the importance of S1P in inflammation signaling and pro-survival pathways. S1P conducts autocrine and paracrine signaling through S1PRs. S1PRs are also highly expressed during inflammation, enhancing S1P signaling to promote pro-inflammatory cytokine secretion, cell survival, and proliferation [37]. S1PRs are involved in the transportation, activation, and cytokine secretion of immune cells, and have been widely studied in intestinal inflammation and cancer. The FDA has approved the selective S1PR1, 4, 5 modulator etrasimod for use in adults with moderate to severe active UC [28]. We found in previous studies that ATX could induce the occurrence and development of colitis in mice [16]. Compared with the control group, ATX-induced mice showed weight loss, colon shortening, increased disease activity, and increased histological scores. In the spleen and mesenteric lymph nodes of ATX colitis mice, the percentage of Th17 cells increased and the percentage of Treg cells decreased. We intervened ATX-induced colitis mice with etrasimod, and found that the colitis symptoms were alleviated, while the changes in Th17/Treg cell balance induced by ATX were partially reversed. Therefore, ATX might induce intestinal inflammation by regulating Th17/Treg cell balance through S1P/S1PRs.

According to reports, ATX is mainly secreted by adipocytes and is widely expressed in a variety of tissues and organs [38]. Our previous research found that in DSS-induced acute colitis mouse models, B cells and macrophages in the colon are the cellular sources of ATX [15]. We applied ATX to HT-29 and Raw264.7, and detected an increase in S1P content and S1PR expression, with S1PR1 showing the most significant increase, confirming the regulatory effect of ATX on S1P/S1PR1. Given these data, it is reasonable to infer that myeloid-derived ATX fuels local S1P, which subsequently acts on T cell S1PR1; direct quantification of subset-derived S1P awaits targeted stable-isotope tracing in future work. Studies have shown that S1P/S1PR1 signaling promotes the differentiation of T cells into Th17 cells [24,39]. The phosphorylation of S1PR1 at the S351 site is necessary for the production of Th17 cells in adaptive immunity [40], and interference with S1PR1 can affect the expression of Th17 cell-related cytokines [41]. In addition, the S1P/S1PR1 pathway exhibits an inhibitory effect on the differentiation of Treg cells [42]. While S1P modulators may affect lymphocyte migration, see Supplementary Figure S2, our absolute cell counting supports a primary role of ATX-S1P/S1PR1 signaling in regulating Th17/Treg differentiation rather than solely affecting lymphocyte trafficking. We validated the promoting effect of ATX on colitis and its regulatory effect on Th17/Treg cell differentiation and function through the S1P/S1PR1 signaling pathway in mice using S1PR1 selective antagonists or agonists. Finally, the S1PR1 selective inhibitor W146 exhibited an alleviating effect on DSS-induced acute colitis, and its combination with the ATX inhibitor showed better therapeutic efficacy.

## 4. Materials and Methods

### 4.1. Patients and Specimens

The blood and colonic tissue specimens of healthy volunteers and UC (ulcerative colitis) patients were sourced from the Department of Gastroenterology, Tongji Hospital, Tongji Medical College, Huazhong University of Science and Technology (from 2020 to 2022). The UC patients involved in the experiment were clinical patients diagnosed according to relevant consensus opinions. All specimens were obtained in accordance with the guidelines of the Declaration of Helsinki and with the informed consent of the participants, and were approved by the Ethics Committee of Tongji Hospital, Tongji Medical College, Huazhong University of Science and Technology (TJ-IRB20201018).

### 4.2. Mice and Experiment

Eighty female C57BL/6 mice aged 6 to 8 weeks (weighing 18 to 22 g) were purchased from Model Organisms Technologies Co., Ltd. (Shanghai, China) and housed in an SPF-level animal facility. The animal experiment operations were carried out with reference to the “Guide for the Care and Use of Laboratory Animals” of the National Institutes of Health in the United States. Approved by the Laboratory Animal Ethics Committee of Tongji Medical College, Huazhong University of Science and Technology (TJH-202208004), all animal experiments were conducted in the Scientific Research Building of Tongji Hospital, Tongji Medical College, Huazhong University of Science and Technology.

Female C57BL/6 mice aged 6 to 8 weeks were selected and randomly divided into 10 groups: the control group (water only), the DSS group (3% DSS), the DSS + PF8380 group (PF8380, ATX inhibitor, 10 mg/kg), the DSS + W146 group (W146, S1PR1 selective antagonist, 10 mg/kg), the DSS + PF8380 + W146 group, the ATX group (50 mg/kg), the ATX + APD334 group (APD334, S1PR1, 4, 5 antagonist, 3 mg/kg), the ATX + PF8380 group, the ATX + W146 group, and the ATX + PF8380 + SEW2871 group (SEW2871, S1PR1 selective agonist, 20 mg/kg). There were 8 mice in each group. The mice were adaptively fed in an SPF animal facility for one week before the establishment of the model.

### 4.3. Assessment of Severity of Colitis

Observe the body weight, fecal characteristics, and bloody stool condition of the mice every day, and calculate the Disease Activity Index (DAI) score of the mice according to Supplementary Table S1. The DAI value is calculated as the sum of the body weight loss index (%), the scores of fecal characteristics, and the scores of bloody stool around the anus, divided by 3. After fixing the colonic tissues of the mice, embedding them in paraffin and slicing, the tissues were stained with hematoxylin-eosin (HE) and observed under a microscope. According to Supplementary Table S2, the histological activity index (HAI) scores were given for the colonic inflammation of the mice.

### 4.4. Cell Culture

The human colon cancer cell line HT-29 and the mouse mononuclear macrophage leukemia cell line Raw264.7 used were stored in our laboratory (Liver and Gastroenterology Laboratory, Tongji Hospital, Tongji Medical College, Huazhong University of Science and Technology). HT-29 cells and Raw264.7 cells were cultured in DMEM medium containing 10% fetal bovine serum (FBS) and 1% penicillin-streptomycin. The incubator conditions were a constant temperature of 37 °C with 5% carbon dioxide.

### 4.5. Quantitative Real-Time RT-PCR Analysis

Total RNA was extracted using TRIzol reagent (Invitrogen, Carlsbad, CA, USA), and the RNA purity was ensured to be maintained within the range of 1.7 < OD260/OD280 < 2. RNA was reverse-transcribed into cDNA using PrimeScript RT Master Mix (Vazyme, Nanjing, China). The analysis was carried out on the QuantStudio 3 real-time fluorescence quantitative PCR system with the SYBR qPCR Master Mix kit (Vazyme, Nanjing, China). Finally, the relative mRNA level was calculated according to the 2^−ΔΔCT^ method. The primer amplification efficiency should be guaranteed to be between 85% and 115%. The primer sequences used are shown in Supplementary Table S3.

### 4.6. Western Blotting

Total protein of tissue and cells was extracted and subjected to Western blot analysis as previously described [43]. The primary antibodies were shown as follows: Alpha Tubulin Antibody (Proteintech, Wuhan, China), S1PR1 Polyclonal Antibody (ImmunoWay, San Jose, CA, USA).

### 4.7. RNA Interference by Small Interfering RNA

For RNA interference, S1PR1-1-siRNA was synthesized by RiboBio company (Guangzhou, China) and then transfected respectively into cells using Lipofectamine 3000 (Invitrogen, Carlsbad, CA, USA).

### 4.8. Flow Cytometry Detection of the Th17/Treg Cell Ratio

Under sterile conditions, the spleen and mesenteric lymph node tissues were isolated, minced, and ground through a sieve to prepare a single-cell suspension. After removing red blood cells with a red blood cell lysis buffer, the cells were washed with RPMI-1640 medium and the cell pellet was collected by centrifugation. Then, cell staining was carried out. First, the cells were incubated with fluorescently labeled antibodies against the surface antigens CD45, CD3, and CD4. After fixing the cells and permeabilizing the membranes, fluorescently labeled antibodies against the intracellular antigens RORγt and FOXP3 were added, followed by thorough washing. Subsequently, the flow cytometer was calibrated using standard microspheres. The stained cell suspension was loaded onto the flow cytometer. After setting parameters such as the laser wavelength and fluorescence channels, the detection was started. The scattered light and fluorescence signals of the cells were collected to complete the data acquisition. Finally, the acquired data were analyzed using FlowJo (v10.8.1) to obtain information such as the proportion of cell subsets and the expression levels of cell-surface or intracellular antigens.

### 4.9. Data Processing

All data were processed using the GraphPad Prism 7.0 software (San Diego, CA, USA) and presented as the mean ± standard error. When comparing statistical differences among more than three groups, one-way analysis of variance (ANOVA) was used to determine the differences between groups. For comparisons involving two groups, Student’s *t*-test was used. A *p*-value < 0.05 was considered statistically significant.

## 5. Conclusions

Our research findings demonstrate that ATX regulates the differentiation and function of Th17/Treg cells through the S1P/S1PR1 signaling pathway, increased ATX affecting Th17/Treg cell balance, leading to intestinal mucosal immune dysfunction and exacerbating intestinal inflammation.

## Data Availability

The original contributions presented in this study are included in the article and Supplementary Materials. Further inquiries can be directed to the corresponding authors.

## Supplementary Materials

The following supporting information can be downloaded at: https://www.mdpi.com/article/10.3390/ijms27062861/s1.

## Abbreviations

The following abbreviations are used in this manuscript:

UC	Ulcerative Colitis
ATX	Autotaxin
S1P	Sphingosine-1-Phosphate
S1PRs	Sphingosine-1-Phosphate Receptors
SPC	Sphingosylphosphorylcholine
