# Supplementary material for: Autotaxin Induces S1P/S1PR1 Signaling to Affect Th17/Treg Cell Balance and Exacerbate Intestinal Inflammation in Colitis
Source: Int J Mol Sci. 2026 Mar 21;27(6):2861. doi: 10.3390/ijms27062861 (PMC13026295; doi:10.3390/ijms27062861)
Supplement: Supplementary file 1 [file ijms-27-02861-s001.zip › ijms-4053628-supplementary.pdf]

## Supplementary Figure S1

**A.**

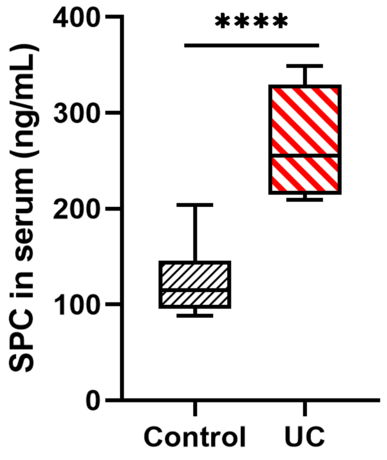


**Supplementary Figure S1.** (A) The secretion level of SPC in the serum of UC patients and healthy volunteers was detected by ELISA. The data are presented as mean ± standard error (n = 10). Compared with the Control group, ****P < 0.0001.

## Supplementary Figure S2

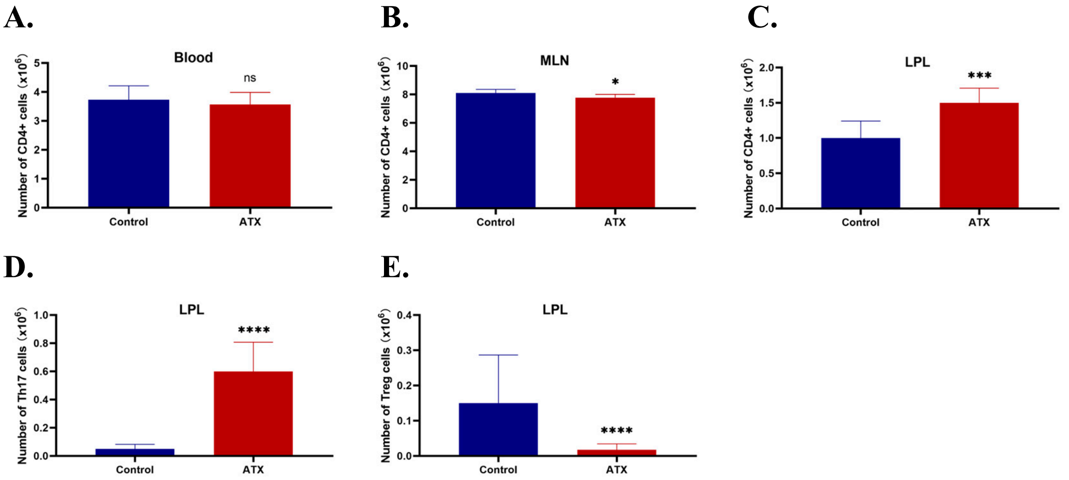


**Supplementary Figure S2.** (A, B, C) Number of $CD4^+$ cells in the blood, MLN, and LPL of mice. (D, E) Number of Th17 & Treg cells in the LPL of mice. The data are presented as mean ± standard error (n = 6). Compared with the Control group, *P < 0.05, ***P < 0.001, ****P < 0.0001.

**Supplementary Table S1. Scoring of disease activity index (DAI)**

| Score | Weight loss (%) | Stool consistency[a] | Occult/gross bleeding |
|---|---|---|---|
| 0 | None | Normal | Normal |
| 1 | 1-5 | | |
| 2 | 5-10 | Loose stools | Hemoccult + |
| 3 | 10-15 | | |
| 4 | ＞15 | Diarrhoea | Gross bleeding |

The DAI value is calculated as the sum of scores of % weight loss, stool consistency and blood in feces

[a] Normal stools: pellets shape; loose: pasty stools, which do not stick to the anus; diarrhoea: liquid stools that stick to the anus

**Supplementary Table S2. Histologic scoring (HAI)**

| Feature scored | Score | Description |
|---|---|---|
| Severity of inflammation | 0 | None |
| | 1 | Mild |
| | 2 | Moderate |
| | 3 | Severe |
| Extent of inflammation | 0 | None |
| | 1 | Mucosa |
| | 2 | Mucosa and submucosa |
| | 3 | Transmural |
| Crypt damage | 0 | None |
| | 1 | Basal 1⁄3 damaged |
| | 2 | Basal 2⁄3 damaged |
| | 3 | Crypts lost; surface epithelium present |
| | 4 | Crypts lost and surface epithelium lost |
| Percentage involvement | 0 | 0% |
| | 1 | 1-25% |
| | 2 | 26-50% |
| | 3 | 51-75% |
| | 4 | 76-100% |

Histologic scores were calculated by multiplying the score for the first three parameters by their percentage involvement, giving a maximum score of 40

**Supplementary Table S3. Primers For RT-qPCR**

| | Forward (5′- 3′) | Reverse (5′- 3′) |
|---|---|---|
| Human-S1PR1 | TTCCACCGACCCATGTACTAT | GCGAGGAGACTGAACACGG |
| Human-S1PR2 | CATCGTCATCCTCTGTTGCG | GCCTGCCAGTAGATCGGAG |
| Human-S1PR3 | CGGCATCGCTTACAAGGTCAA | GCCACGAACATACTGCCCT |
| Human-S1PR4 | GACGCTGGGTCTACTATTGCC | CCTCCCGTAGGAACCACTG |
| Human-S1PR5 | GCGCACCTGTCCTGTACTC | GTTGGTGAGCGTGTAGATGATG |
| Human-TNF-α | CCTCTCTCTAATCAGCCCTCTG | GAGGACCTGGGAGTAGATGAG |
| Human-IL-1β | TTCGACACATGGGATAACGAGG | TTTTTGCTGTGAGTCCCGGAG |
| Human-IL-6 | CCTGAACCTTCCAAAGATGGC | TTCACCAGGCAAGTCTCCTCA |
| Human-IL-17A | TCCCACGAAATCCAGGATGC | GGATGTTCAGGTTGACCATCAC |
| Human-IL-10 | TCAAGGCGCATGTGAACTCC | GATGTCAAACTCACTCATGGCT |
| Human-STAT3 | CAGCAGCTTGACACACGGTA | AAACACCAAAGTGGCATGTGA |
| Human-RORγt | GTGGGGACAAGTCGTCTGG | AGTGCTGGCATCGGTTTCG |
| Human-FOXP3 | GTGGCCCGGATGTGAGAAG | GGAGCCCTTGTCGGATGATG |
| Human-GAPDH | GGAGTCCACTGGCGTCTTCA | GTCATGAGTCCTTCCACGATACC |
| Mouse-S1PR1 | ATGGTGTCCACTAGCATCCC | CGATGTTCAACTTGCCTGTGTAG |
| Mouse-S1PR2 | ACAGCAAGTTCCACTCAGCAA | CTGCACGGGAGTTAAGGACAG |
| Mouse-S1PR3 | ACTCTCCGGGAACATTACGAT | CCAAGACGATGAAGCTACAGG |
| Mouse-S1PR4 | CTGGCTACTGGCAGCTATCC | AGACCACCACACAAAAGAGCA |
| Mouse-S1PR5 | GCTTTGGTTTGCGCGTGAG | GGCGTCCTAAGCAGTTCCAG |

| | | |
|---|---|---|
| Mouse-TNF-α | CCCTCACACTCACAAACCAC | ACAAGGTACAACCCATCGGC |
| Mouse-IL-1β | GAAATGCCACCTTTTGACAGTG | TGGATGCTCTCATCAGGACAG |
| Mouse-IL-6 | CCAAGAGGTGAGTGCTTCCC | CTGTTGTTCAGACTCTCTCCCT |
| Mouse-IL-17A | TTTAACTCCCTTGGCGCAAAA | CTTTCCCTCCGCATTGACAC |
| Mouse-IL-10 | GCTGGACAACATACTGCTAACC | ATTTCCGATAAGGCTTGGCAA |
| Mouse-STAT3 | CAATACCATTGACCTGCCGAT | GAGCGACTCAAACTGCCCT |
| Mouse-RORγt | GACCCACACCTCACAAATTGA | AGTAGGCCACATTACACTGCT |
| Mouse-FOXP3 | CCCATCCCCAGGAGTCTTG | ACCATGACTAGGGGCACTGTA |
| Mouse-GAPDH | CAGTGGCAAAGTGGAGATTGTTG | TCGCTCCTGGAAGATGGTGAT |
